# Rapid syndromic PCR testing in patients with respiratory tract infections reduces time to results and improves microbial yield

**DOI:** 10.1038/s41598-021-03741-7

**Published:** 2022-01-10

**Authors:** S. Serigstad, D. Markussen, H. M. S. Grewal, M. Ebbesen, Ø. Kommedal, L. Heggelund, C. H. van Werkhoven, D. Faurholt-Jepsen, T. W. Clark, C. Ritz, E. Ulvestad, R. Bjørneklett, S. T. Knoop, The CAPNOR Study Group, R. Bjørneklett, T. W. Clark, M. Ebbesen, D. Faurholt-Jepsen, H. M. S. Grewal, L. Heggelund, S. T. Knoop, Ø. Kommedal, D. Markussen, P. Ravn, C. Ritz, S. Serigstad, E. Ulvestad, C. H. van Werkhoven

**Affiliations:** 1 Emergency Care Clinic, Haukeland University Hospital, Bergen, Norway; 2 Department of Clinical Medicine, University of Bergen, Bergen, Norway; 3 Department of Clinical Science, Bergen Integrated Diagnostic Stewardship Cluster, Faculty of Medicine and Dentistry, University of Bergen, The New Lab. Building, NO-5021 Bergen, Norway; 4 Department of Microbiology, Haukeland University Hospital, Bergen, Norway; 5 Department of Internal Medicine, Vestre Viken Hospital Trust, Drammen, Norway; 6 Julius Center for Health Sciences and Primary Care, University Medical Center Utrecht, Utrecht, The Netherlands; 7 Department of Infectious Diseases, Rigshospitalet, Copenhagen, Denmark; 8 School of Clinical and Experimental Sciences, Faculty of Medicine, University of Southampton, Southampton, UK; 9 National Institute of Public Health, University of Southern Denmark, Copenhagen, Denmark; 10 Section for Infectious Diseases, Department of Internal Medicine, Herlev and Gentofte Hospital, Hellerup, Denmark

**Keywords:** Bacterial infection, Viral infection, Respiratory tract diseases, Clinical microbiology, Infectious-disease diagnostics

## Abstract

Lack of rapid and comprehensive microbiological diagnosis in patients with community acquired pneumonia (CAP) hampers appropriate antimicrobial therapy. This study evaluates the real-world performance of the BioFire FilmArray Pneumonia panel *plus* (FAP *plus*) and explores the feasibility of evaluation in a randomised controlled trial. Patients presenting to hospital with suspected CAP were recruited in a prospective feasibility study. An induced sputum or an endotracheal aspirate was obtained from all participants. The FAP *plus* turnaround time (TAT) and microbiological yield were compared with standard diagnostic methods (SDs). 96/104 (92%) enrolled patients had a respiratory tract infection (RTI); 72 CAP and 24 other RTIs. Median TAT was shorter for the FAP *plus*, compared with in-house PCR (2.6 vs 24.1 h, p < 0.001) and sputum cultures (2.6 vs 57.5 h, p < 0.001). The total microbiological yield by the FAP *plus* was higher compared to SDs (91% (162/179) vs 55% (99/179), p < 0.0001). *Haemophilus influenzae*, *Streptococcus pneumoniae* and influenza A virus were the most frequent pathogens. In conclusion, molecular panel testing in adults with CAP was associated with a significant reduction in time to actionable results and increased microbiological yield. The impact on antibiotic use and patient outcome should be assessed in randomised controlled trials.

## Introduction

Lower respiratory tract infections (LRTIs), including community acquired pneumonia (CAP), are one of the leading causes of death and years of life lost globally^1,2^. The microbiological etiology of CAP is often difficult to ascertain, due to difficulties in obtaining representative respiratory specimens and insufficient methods for microbial detection^3–5^. Empiric antimicrobial therapy is often initiated in the primary care setting, further hampering diagnostic attempts based on conventional bacterial culture in hospitalised patients. In Norway, data on the etiology of CAP in hospitalised patients is limited to a few studies, which do not include a comprehensive assessment of viral and bacterial respiratory pathogens^6,7^.

Polymerase chain reaction (PCR) based assays targeting respiratory viruses and atypical bacteria have widened our understanding of CAP etiology, and CAP is no longer considered to be exclusively of bacterial origin^3,8–13^. Improved detection of viruses could potentially reduce unnecessary use of antibiotics, although coinfections can be difficult to rule out owing to the lack of comparable methods for the detection of bacterial pathogens. Recently, rapid syndromic PCR panels for viruses and bacteria involved in CAP have been developed. Several studies have evaluated the diagnostic performance of these panels, concluding that they are highly accurate and detect pathogens in a higher proportion compared with standard diagnostic methods (SDs)^14–19^. However, reports on the incorporation of syndromic tests in clinical practice are scarce, and there is limited evidence of the clinical impact of these tests.

Our primary objective was to explore the feasibility of introducing the BioFire FilmArray Pneumonia panel *plus* (FAP *plus*) (bioMérieux S.A., Marcy-l’Etoile, France) in the diagnostic workup of patients admitted with suspected CAP, with a view to inform the design of a subsequent randomised controlled trial. We hypothesized that syndromic PCR-based testing of lower respiratory tract samples is achievable in acute settings. The secondary objectives were to investigate the real-world turnaround time (TAT) and microbiological yield of the FAP *plus* compared to SDs at our hospital.

## Methods

### Patients and study design

This was a prospectively recruited cohort study conducted at Haukeland University Hospital, a tertiary care referral centre in Bergen, Norway between December 2nd 2019, and February 17th 2020. In addition to conventional microbiological diagnostics, samples from the lower respiratory tract were systematically analysed with a commercial rapid syndromic PCR panel, the FAP *plus*. The study was conducted as a feasibility study to inform the design of a larger randomised controlled trial evaluating the clinical impact of the FAP *plus* assay on antibiotic use and outcome (NCT04660084). Patients were eligible for inclusion if they were ≥ 18 years, presenting to the emergency department (ED) with a suspicion of CAP (evaluated by investigating physicians and/or study nurses) and fulfilling at least two of the following criteria: new or worsening cough; new or worsening expectoration of sputum; new or worsening dyspnoea; haemoptysis; pleuritic chest pain; radiological evidence of pneumonia; abnormalities on chest auscultation and/or percussion; fever (≥ 38.0 °C). Exclusion criteria were cystic fibrosis, severe bronchiectasis (defined as patients in need of regular follow-up and treatment by a pulmonologist due to bronchiectasis), hospitalisation within the last 14 days prior to admission, a palliative approach (defined as life expectancy below two weeks documented by a treating physician; either by preexisting estimates in the electronic journal, or estimations made at admission), or if the patient was not willing or able to provide a lower respiratory tract sample (by either sputum induction or endotracheal aspiration).

### Data collection and sampling

Patients were enrolled on weekdays between 08:00 a.m. and 09:00 p.m. Most patients were enrolled in the ED shortly after admission. Investigating physicians and/or study nurses screened all electronic triage documents. Patients with respiratory complaints and/or a suspected infection of any type were then evaluated for eligibility, according to the inclusion criteria. To compensate for the restricted study operating hours, some cases were included at the wards up to a maximum of 24 h after admission. These patients were identified through retrospective screening of electronic triage documents and medical records. Relevant baseline information was collected by study nurses or investigating physicians through a structured interview. Symptoms and findings upon clinical examinations were recorded. Data pertaining to treatment and results from laboratory tests and medical imaging were obtained from electronic medical records and charts. Data were registered in an electronic case report form (eCRF) from VieDoc (Viedoc Technologies, Uppsala, Sweden).

The final diagnosis (as per the four different categories enumerated below) was determined retrospectively, following patient discharge, by the use of pre-specified diagnostic criteria in consensus meetings among investigating physicians. The diagnostic criteria thus differed from the inclusion criteria, which were designed to be used as a screening tool based on the available information at admission in the ED. The complete definitions are provided in the Supplementary material (S2). Patients were categorized into four different categories: (1) Confirmed CAP (with radiological confirmation); (2) Clinical CAP (without radiological confirmation); (3) Other respiratory tract infections (RTIs), i.e., bronchitis, acute infectious exacerbation in patients with chronic obstructive pulmonary disease (COPD), acute infectious exacerbation of asthma, and upper RTIs; or (4) Other diagnosis, i.e., CAP suspected at admission but RTI later disproved. To avoid observer bias an independent agreement between the treating physicians and the study investigators was desired. If any disagreement, an additional study investigator would arbitrate.

### Microbiological sampling and methods

At inclusion, a lower respiratory tract sample for the FAP *plus* and standard culture was obtained from all patients. Depending on clinical symptoms, vital signs and medical history, sputum induction with either nebulized isotonic (0.9%) or hypertonic (5.8%) saline was attempted. Patients with known obstructive lung disease and patients with hypoxemia or signs of airway obstruction upon physical examination, were additionally treated with a bronchodilator (salbutamol and/or ipratropium bromide) prior to sampling. If sputum induction was unsuccessful, endotracheal aspiration was performed. The detailed procedures are provided in the Supplementary material (S3).

The FAP *plus* is an automated multiplex PCR test for the detection of 27 bacteria and viruses, as well as seven genetic markers of antibiotic resistance, validated for lower respiratory tract samples (Supplementary material, S1). The hands-on time is around two minutes and the total analysis time about 1 h^20^. Typical bacterial detections are reported in a semi-quantitative manner and categorized as negative if ≤ 10^3.5^ copies/ml. Above this level, results are reported as positive and semi-quantitatively specified as 10^4^, 10^5^, 10^6^ or ≥ 10^7^ copies/ml^21^.

The SDs included culture of respiratory tract samples and blood according to current guidelines (adapted from^22^). Blood culture isolates and relevant respiratory isolates were identified with matrix-assisted laser desorption/ionization time-of-flight mass spectrometry (MALDI-ToF MS) using the Bruker's microflex LT instrument, MBT Compass software ver. 4.1 and Compass Library DB-8468 (Bruker Daltonics, Massachusetts, U.S.). Nasopharyngeal and/or oropharyngeal swabs were examined by an in-house real-time PCR test to detect respiratory viruses and atypical bacteria (influenza A and B, human parainfluenza viruses 1–3, respiratory syncytial virus, human metapneumovirus, rhinovirus, *Bordetella pertussis*, *Bordetella parapertussis*, *Mycoplasma pneumoniae* and *Chlamydia pneumoniae*). SDs also included rapid diagnostic tests; the pneumococcal urine antigen test (Quidel Corporation, San Diego, U.S.) and a point of care (POC) test for influenza virus A and B (ID NOW, Illinois, U.S.). Any additional tests requested by the treating physician were also noted and counted as part of SDs.

Gram staining was utilized to evaluate the representativeness of all sputum samples (adapted from^22^). Samples containing ≥ 10 squamous epithelial cells per field in at least 10 fields with 10 × enlargement were considered non-representative. However, this criterion was disregarded if a significant amount of both leukocytes (≥ 10 times the amount of squamous epithelial cells per field of view) and a morphologically uniform microbe (> 5 microbes per field of view with 100× enlargement) were present. All samples were analysed by the FAP *plus* and cultured on agar-plates, irrespective of the representativeness. Abundant growth of plausible respiratory pathogens was reported regardless of the representativeness of the sputum sample. Non-abundant growth was only reported in samples considered representative.

The clinical relevance of all microbiological findings, in terms of categorization as a relevant pathogen for the current RTI or not, was established retrospectively using pre-specified criteria (Supplementary material, S4).

### Statistical analysis

Descriptive statistics for continuous variables are reported as median with interquartile range (IQR). Turn-around time for microbiological methods are compared with Student’s paired t-test on logarithm-transformed times. For paired categorical samples, McNemar’s test with risk differences and 95% confidence intervals (CIs) are used. A two tailed p-value ≤ 0.05 was considered statistically significant for all analyses. The statistics were performed using IBM SPSS Statistics (version 26.0; Armonk, NY, U.S.), the statistical environment R (version 3.6.3; Vienna, Austria) and the GraphPad QuickCalcs Web site: http://www.graphpad.com/quickcalcs/McNemar1.cfm (last accessed 18. March 2021).

### Ethics

The study was approved by the Regional Committee for Medical and Health Research Ethics in South-Eastern Norway (REK ID: 31935) and performed in accordance with the Declaration of Helsinki. Written informed consent was obtained from all participants or from their legal guardian/close relative at the time of recruitment.

## Results

### Patient characteristics

**Figure 1:**
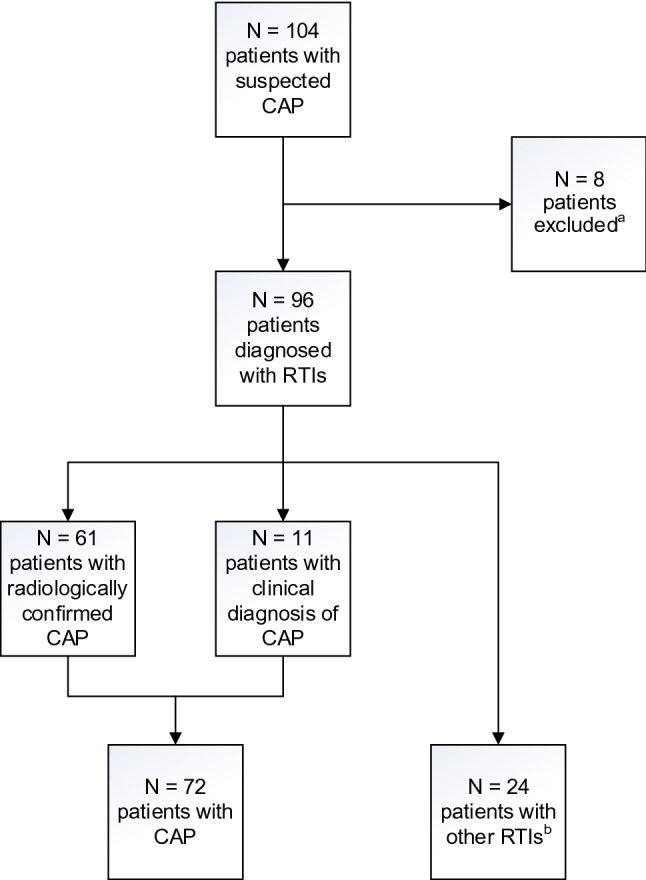
Study flowchart. CAP, community acquired pneumonia; RTI, respiratory tract infection; COPD, chronic obstructive pulmonary disease. ^a^Eight patients were excluded due to other diagnoses: non-infectious exacerbation of COPD (n = 2); heart failure (n = 2); unspecified non-infectious dyspnea (n = 2); urinary tract infection (n = 1); aortic graft infection (n = 1). ^b^Acute bronchitis (n = 10), acute infectious exacerbation of COPD (n = 8), acute infectious exacerbation of asthma (n = 4) and upper RTI (n = 2).

A total of 104 patients with suspected CAP were enrolled (Fig. 1). Eight patients were subsequently diagnosed with an alternative diagnosis (non-infectious exacerbation of COPD (n = 2), hearth failure (n = 2), unspecified non-infectious dyspnoea (n = 2), urinary tract infection (n = 1), aortic graft infection (n = 1)), and were excluded from further analyses. Baseline characteristics for the final cohort of 96 patients with an RTI are shown in Table 1. The majority of patients (69/96 (72%)) were recruited in the ED, with a median (IQR) time of 31 (18–77) minutes from hospital admission to enrolment. For the 27 (28%) patients recruited in the wards, the median (IQR) time to enrolment was 17 (15–21) hours. Overall, 61 (64%) patients had a radiologically confirmed CAP, 11 (11%) patients had CAP without radiological confirmation, and 24 (25%) patients had other RTIs (acute bronchitis (n = 10), acute infectious exacerbation of COPD (n = 8), acute infectious exacerbation of asthma (n = 4) and upper RTI (n = 2)). In the following sections, radiological confirmed CAP and clinically diagnosed CAP without radiological confirmation are considered as a single entity (n = 72).

**Table 1:** Characteristics of the study cohort of n = 96 patients with community acquired respiratory tract infections.

	CAP (n = 72)	Other RTIs (n = 24)
**A: Baseline characteristics**		
Demography		
Age	74 (61–81)	65 (51–75)
Male	35 (49)	9 (38)
Comorbidity		
Cardiovascular disease	38 (53)	10 (42)
Diabetes mellitus	5 (7)	6 (25)
Asthma/COPD	26 (36)	12 (50)
Kidney disease	12 (17)	3 (13 )
Previous smoker	37 (51)	6 (25)
Current smoker	10 (14)	10 (42)
Vaccine status^a^		
Influenza virus	42 (58)	16 (67)
Pneumococcal	17 (24)	6 (25)
**B: Severity and outcome**		
Biochemistry^b^		
WBC count	13.7 (9.3–16.6)	9.5 (7.1–10.9)
CRP level	175 (112–250)	57 (34–82)
Severity score^c^		
CURB-65	1.0 (1.0–2.0)	–
PSI^d^	93 (71–111)	–
Outcome		
Length of stay (days)	3.6 (2.2–5.2)	1.9 (0.4–3.2)
HDU or ICU admission	6 (8.3)	4 (16.7)
Case fatality rate		
In-hospital	1 (1)	0 (0)
30 days	1 (1)	0 (0)
60 days	4 (6)	0 (0)

Data shown as count (%) or median (IQR).

CAP, community acquired pneumonia; RTI, respiratory tract infection; COPD, chronic obstructive pulmonary disease; WBC, white blood cells; CRP, C-reactive protein; CURB-65, confusion, urea, respiratory rate, blood pressure, age ≥ 65 years; PSI, pneumonia severity index; HDU, high dependency unit; ICU, intensive care unit; IQR, interquartile range.

^a^Vaccinated for influenza virus within the last year; for *Streptococcus pneumoniae* within the last 5 years.

^b^Highest value during hospital stay.

^c^Only validated for CAP patients.

^d^Missing in five patients with CAP.

Respiratory tract specimens were obtained in all RTI-patients, mainly by sputum induction (91/96 (95%)), the remaining by endotracheal aspiration (5/96 (5%)). By Gram-staining, 56 of 96 (58%) respiratory tract samples were classified as not representative of the lower respiratory tract. All samples were investigated using the FAP *plus* rapid PCR panel in addition to standard culture-based methods. Blood cultures were performed in 95 (99%), in-house PCR testing in 87 (91%), the POC influenza test in 76 (79%), and a pneumococcal urine antigen test in 62 (65%) of the patients.

### Time to results

The TAT, i.e., time from sampling to a reported test result, varied considerably by method (Table 2). Median TAT for the FAP *plus* was strikingly shorter, compared with in-house PCR (2.6 vs 24.1 h, median difference of 21.0 h (IQR 16.3–24.7), p < 0.001) and sputum cultures (both negative and positive combined) (2.6 vs 57.5 h, median difference of 48.6 h (IQR 24.0–91.5), p < 0.001). Further, results from sputum cultures were not reported until antimicrobial susceptibility testing (AST) was completed, implying an even larger difference in TAT when the FAP *plus* was compared with solely positive sputum cultures (2.6 vs 92.6 h, median difference of 89.1 h (IQR 47.4–130.2), p < 0.001).

**Table 2:** Turnaround time for the BioFire FilmArray Pneumonia panel *plus* versus standard microbiological methods.

Diagnostic method	Patients (n)	Turnaround time	Time difference^a^	p-value^a^
FAP *plus*	96	2.6 (2.2–3.4)	N.A	N.A
In-house PCR	87	24.1 (19.6–27.8)	21.0 (16.3–24.7)	< 0.001
Sputum culture	96	57.5 (26.9–94.4)	48.6 (24.0–91.5)	< 0.001
Pneumococcal antigen	62	1.3 (0.9–1.8)	− 1.2 (− 2.0 to (− 0.5))	< 0.001
POC influenza^b^	76	–^b^	–	–

Comparison of the turnaround time for different microbiological diagnostic methods used in this study. Numbers presented are median hours with IQR if not otherwise specified. P-values are calculated with Student’s paired t-test on logarithm-transformed times.

FAP *plus*, Biomérieux BioFire FilmArray Pneumonia panel *plus*; PCR, polymerase chain reaction; POC, point of care; N.A., not applicable; IQR, inter-quartile range.

^a^Compared to the FAP *plus.*

^b^Turnaround time was not recorded for the POC Influenza test (ID NOW). It was directly available in the ED, with an analysis time of approximately 15 min.

### Microbiological findings

Eighty-nine out of the 96 (93%) RTI-patients had at least one positive microbiological test, yielding a total of 179 microbes. A considerable proportion of the microbes, 64 of 179 (36%), were deemed to be of uncertain relevance. The remaining 115 (64%) microbes were deemed clinically relevant pathogens and were recovered from 83 (86%) patients, including 61 of the 72 (85%) CAP patients. Among the total CAP patients, 24 (33%) had pure bacterial detections, 23 (32%) bacterial and viral co-detections, 13 (18%) pure viral detections, 11 (15%) had no detections, while one (1%) was diagnosed with a *Pneumocystis jirovecii* infection. *Haemophilus influenzae* (32% (23/72)), *Streptococcus pneumoniae* (28% (20/72)) and influenza A virus (22% (16/72)) were the most frequently detected pathogens. More than one relevant microbe was detected in 35% (25/72) of CAP patients. Among the 24 patients with other RTIs, a viral etiology was detected in 92% (22/24) of patients, most frequently influenza A virus (54% (13/24)).

### Comparison of the FAP *plus* to SDs

Except for three cases of human metapneumovirus, all microbes found by the in-house PCR test were also detected by the FAP *plus*. A relevant detection of *Staphylococcus aureus* by blood culture, two positive *S. pneumoniae* urinary antigen tests and a case of *P. jirovecii* were solely made by SDs. The complete microbiological findings by the FAP *plus* versus SDs are listed in Tables 3 and 4, stratified by evaluation of relevance and diagnostic category. The total microbiological yield was significantly higher by use of the FAP *plus* compared to SDs (91% (162/179) vs 55% (99/179), difference of 35% (95%CI 25–45%, p < 0.0001)). Using the FAP *plus* affected both the proportion of relevant, assumed causative detections (94% (108/115) vs 69% (79/115), difference of 25% (95%CI 14–36%, p < 0.0001)), and the proportion of detections with uncertain relevance (84% (54/64) vs 31% (20/64), difference of 53% (95%CI 33–73%, p < 0.0001)). In CAP patients included at the wards (n = 23), where 21 (91%) had received antibiotic treatment before the collecting of respiratory specimens, the FAP *plus* detected 92% (12/13) of relevant bacterial pathogens compared to 31% (4/13) by SDs (difference of 62% (95%CI 20–103%, p = 0.0269)). For the CAP patients enrolled in the ED (n = 49), the FAP *plus* detected 95% (37/39) of relevant bacterial pathogens compared to 64% (25/39) by SDs (difference of 31% (95%CI 11–51%, p = 0.006)).

**Table 3:** Microbiological findings by use of the BioFire FilmArray Pneumonia panel *plus* versus standard microbiological methods in 72 patients with CAP.

Microbes	Community acquired pneumonia (CAP)					
	Total detections*			Detections deemed ^a^ relevant**		
	All methods combined	FAP *plus*	Standard methods	All methods combined	FAP *plus*	Standard methods
**Viruses**	38	35 (92)	28 (74)	38	35 (92)	28 (74)
Influenza A virus	16	16 (100)	13 (81)	16	16 (100)	13 (81)
Human metapneumovirus	13	10 (77)	10 (77)	13	10 (77)	10 (77)
Respiratory syncytial virus	3	3 (100)	3 (100)	3	3 (100)	3 (100)
Coronavirus	3	3 (100)	–	3	3 (100)	–
Parainfluenza virus	2	2 (100)	2 (100)	2	2 (100)	2 (100)
Rhino-/enterovirus	1	1 (100)	0 (0)	1	1 (100)	0 (0)
**Bacteria**	91	**81 (89)**	41 (45)	52	**49 (94)**	30 (58)
*H. influenzae*	25	**25 (100)**	14 (56)	23	**23 (100)**	13 (57)
*S. pneumoniae*	20	18 (90)	11 (55) ^b^	20	18 (90)	11 (55) ^b^
*M. catarrhalis*	8	**8 (100)**	2 (25)	2	2 (100)	2 (100)
*E. coli*	6	5 (83)	2 (33)	0	0 (–)	0 (–)
*S. aureus*	5	4 (80)	2 (40)	2	1 (50)	1 (50) ^c^
*S. agalactiae*	5	5 (100)	0 (0)	0	0 (–)	0 (–)
*S. epidermidis*	5	–	5 (100)	0	–	0 (–)
*K. pneumoniae*	3	3 (100)	1 (33)	1	1 (100)	0 (0)
*S. marcescens*	3	3 (100)	1 (33)	1	1 (100)	1 (100)
*P. aeruginosa*	3	2 (67)	1 (33)	0	0 (–)	0 (–)
*Proteus* spp.	2	2 (100)	0 (0)	0	0 (–)	0 (–)
*K. oxytoca*	1	1 (100)	0 (0)	0	0 (–)	0 (–)
*A. calcoaceticus*–*A. baumanii* complex	1	1 (100)	0 (0)	0	0 (–)	0 (–)
*E. cloacae complex*	1	1 (100)	0 (0)	0	0 (–)	0 (–)
*M. pneumoniae*	2	2 (100)	1 (50)	2	2 (100)	1 (50)
*L. pneumophila*	1	1 (100)	1 (100)^d^	1	1 (100)	1 (100)^d^
**Other detections**	4	–	4 (100)	1	–	1 (100)
*C. albicans*	3	–	3 (100)	0	–	0 (–)
*P. jirovecii*	1	–	1 (100)	1	–	1 (100)
**Total**	133	**116 (87)**	73 (55)	91	**84 (92)**	59 (65)

Microbiological findings provided by the syndromic PCR panel (FAP *plus*) compared to SDs in CAP patients (n = 72). Detections deemed as clinically relevant ^a ^pathogens are further specified. *Data are shown as number of detections with percentage of the respective microbe’s total detections (*All methods combined*) in brackets. **Data are shown as number of relevant detections with percentage of the respective microbe’s total relevant detections (*All methods combined*) in brackets. Statistically significant differences (McNemar’s test, p < 0.05) between the FAP *plus* and SDs, are marked with bold fonts.

CAP, community acquired pneumonia; FAP *plus*, Biomérieux BioFire FilmArray Pneumonia panel *plus*; SDs, standard diagnostic methods; –, not applicable; FIA, fluorescent immunoassay.

^a^The clinical relevance of all detected microbes was evaluated retrospectively using pre-specified criteria (Supplementary material, S4).

^b^Sputum culture detected five patients with *S. pneumoniae*. A pneumococcal antigen detection test in urine (Sofia *S. Pneumoniae* FIA, Quidel) was positive in ten patients, of which five were unique findings, whereas one was in combination with a positive blood culture.

^c^Detected in blood culture only.

^d^*Legionella pneumophila* antigen detection test in urine (Sofia Legionella FIA, Quidel).

**Table 4:** Microbiological findings by use of the BioFire FilmArray Pneumonia panel *plus* versus standard microbiological methods in 24 patients with other respiratory tract infections.

Microbes	Other respiratory tract infections (other RTIs)					
	Total detections*			Detections deemed^a^ relevant**		
	All methods combined	FAP *plus*	Standard methods	All methods combined	FAP *plus*	Standard methods
**Viruses**	24	24 (100)	20 (83)	24	24 (100)	20 (83)
Influenza A virus	13	13 (100)	13 (100)	13	13 (100)	13 (100)
Human metapneumovirus	3	3 (100)	2 (67)	3	3 (100)	2 (67)
Respiratory syncytial virus	4	4 (100)	3 (75)	4	4 (100)	3 (75)
Coronavirus	2	2 (100)	–	2	2 (100)	–
Parainfluenza virus	0	0 (–)	0 (–)	0	0 (–)	0 (–)
Rhino-/enterovirus	2	2 (100)	2 (100)	2	2 (100)	2 (100)
**Bacteria**	22	**22 (100)**	6 (27)	0	0 (–)	0 (–)
*H. influenzae*	10	**10 (100)**	4 (40)	0	0 (–)	0 (–)
*S. pneumoniae*	4	4 (100)	0 (0)	0	0 (–)	0 (–)
*M. catarrhalis*	3	3 (100)	1 (33)	0	0 (–)	0 (–)
*E. coli*	2	2 (100)	0 (0)	0	0 (–)	0 (–)
*S. aureus*	2	2 (100)	1 (50)	0	0 (–)	0 (–)
*S. agalactiae*	1	1 (100)	0 (0)	0	0 (–)	0 (–)
*S. epidermidis*	0	–	0 (–)	0	–	0 (–)
*K. pneumoniae*	0	0 (–)	0 (–)	0	0 (–)	0 (–)
*S. marcescens*	0	0 (–)	0 (–)	0	0 (–)	0 (–)
*P. aeruginosa*	0	0 (–)	0 (–)	0	0 (–)	0 (–)
*Proteus spp.*	0	0 (–)	0 (–)	0	0 (–)	0 (–)
*K. oxytoca*	0	0 (–)	0 (–)	0	0 (–)	0 (–)
*A. calcoaceticus*–*A. baumanii* complex	0	0 (–)	0 (–)	0	0 (–)	0 (–)
*E. cloacae complex*	0	0 (–)	0 (–)	0	0 (–)	0 (–)
*M. pneumoniae*	0	0 (–)	0 (–)	0	0 (–)	0 (–)
*L. pneumophila*	0	0 (–)	0 (–)	0	0 (–)	0 (–)
**Other detections**	0	–	0 (–)	0	–	0 (–)
*C. albicans*	0	–	0 (–)	0	–	0 (–)
*P. jirovecii*	0	–	0 (–)	0	–	0 (–)
**Total**	46	**46 (100)**	26 (57)	24	24 (100)	20 (83)

Microbiological findings provided by the syndromic PCR panel (FAP *plus*) compared to SDs in patients with other RTIs (n = 24). Detections deemed as clinically relevant ^a^ pathogens are further specified. *Data are shown as number of detections with percentage of the respective microbe’s total detections (*All methods combined*) in brackets. **Data are shown as number of relevant detections with percentage of the respective microbe’s total relevant detections (*All methods combined*) in brackets. Statistically significant differences (McNemar’s test, p < 0.05) between the FAP *plus* and SDs, are marked with bold fonts.

RTI, respiratory tract infection; FAP *plus*, Biomérieux BioFire FilmArray Pneumonia panel *plus*; SDs, standard diagnostic methods; –, not applicable; FIA, fluorescent immunoassay.

^a^The clinical relevance of all detected microbes was evaluated retrospectively using pre-specified criteria (Supplementary material, S4).

## Discussion

This prospective study is one of the first to evaluate the real-world performance and time to results of a rapid syndromic PCR panel in patients presenting to hospital with suspected CAP. We demonstrated that the routine collection of lower respiratory tract specimens (induced sputum or endotracheal aspirates) in the ED was feasible and found a large improvement in both time to microbiological results and the microbiological yield by use of FAP *plus* compared to SDs. Study enrolment and collection of specimens were in general performed before medical imaging and laboratory results were available. Our patient population is therefore confined to suspected rather than confirmed CAP patients, with the rationale to maximize the potential impact on initial diagnostics and treatment decisions, and reflect actual clinical practice.

During the last decade, research on the etiology of CAP has often included PCR-based methods, although usually limited to a restricted selection of bacterial targets or specimens from the upper respiratory tract^3,6,23,24^. One of the most comprehensive studies is a British investigation of sputum from 323 patients with CAP^8^. Like our results, a pathogen was identified in 87% of patients representing a substantial improvement compared to standard diagnostic testing (30–40%)^3,7^. However, the British study analysed frozen samples retrospectively with resource-consuming in-house PCR procedures. A short analysis time and ease of use are major advantages of automated commercial PCR panels. When embedded in routine clinical practice, we observed a real-world median TAT of 2.6 h for the FAP *plus*, considerably faster than the standard in-house PCR test and sputum culture (Table 2). A direct consequence of a rapid TAT is the provision of near real-time information to treating physicians. This study thus demonstrates a promising potential for informed initial decisions on antimicrobial treatment and isolation. Still, further exploration is needed, preferably in a larger randomised controlled trial, before implementation as a routine test can be considered.

The most frequent pathogens in our CAP cohort were *H. influenzae* and *S. pneumoniae*, a finding similar to that in other studies^6,8,17,24–27^. In Norway, monotherapy with benzylpenicillin, 1.2 g every six hours, is the current empirical treatment recommendation for mild and moderate hospitalised CAP patients. The high prevalence of *H. influenzae* is consistent with previous PCR-based studies and needs further exploration, moreover, EUCAST has stated that there is still insufficient data for *H. influenzae* to set clinical breakpoints for benzylpenicillin^28^. Viral pathogens were also frequently detected in our CAP cohort, in agreement with other studies influenced by winter enrolment^6,23^. The growing recognition of an important role for viruses in CAP is thus supported by our work^3,9–13^.

Samples from the lower respiratory tract are often difficult to obtain in a clinically meaningful time frame. Previously reported collection rates of sputum range from 30 to 60%^3,6,13,23^ and this represents a major barrier to introducing rapid molecular diagnostics for CAP. Induced sputum was obtained from the majority of included patients (95%) in this study, underscoring that successful collection of this material is feasible, well tolerated and achievable in an ED setting. By traditional microscopic criteria, 42% of specimens were considered representative, a rate not different from other reports^3,6,23^. These microscopic criteria have been developed as a pragmatic quality check of samples in traditional culture-based diagnostics. Modern PCR methods can detect and quantify potentially pathogenic bacteria from complex background microbial populations. In this context, the value of pre-analytic microscopy remains uncertain^29^. Detections of viruses and atypical bacteria in RTI-patients are generally considered pathogenic^3,10,12,30–32^. Some data supports that detection of higher quantities of bacterial pathogens are rare among adults without RTI^10,13,31,32^; but this is disputed, especially in patients with chronic obstructive pulmonary disease^33,34^. Intended to aid in the differentiation between colonising bacteria and causative agents, the FAP *plus* provides semi-quantitative information on copy numbers for the bacterial pathobionts. Like others, we chose not to consider semi-quantitative values in our study, because of limited evidence from prior use, variable impact on antimicrobial treatment, and the possible influence of different comorbidities on patterns of colonisation and susceptibility to infections^18^. The correlation between semi-quantitative bacterial PCR levels and clinical relevance requires further exploration in larger settings.

Recent studies reporting on the performance of FAP *plus* also show a considerable increase in bacterial detections compared to SDs^14–19^. However, important limitations include heterogeneous patient populations (random combinations of community-, hospital-, and/or ventilator acquired pneumonia, as well as other RTIs), various sample materials (sputum, tracheal aspirates or broncho-alveolar lavage) and inconsistent testing by SDs^14–17,19^. Clinical data are often scarce, and incompletely accounted for. Therefore, the relevance of many of the detected microbes is uncertain. Evaluation of the clinical relevance of all microbiological results is thus a major strength of this study. Only one other recent FAP *plus* study in CAP patients has made an attempt to determine potential colonisers^18^. Unlike our study, their algorithm included an evaluation of procalcitonin levels to determine relevance and category of infection. The utility of procalcitonin to distinguish viral from bacterial infections is, however, uncertain^35^. Anyhow, determining relevance is difficult, and although not being causative per se*,* microbes of uncertain relevance could still contribute to an ongoing infectious process. Information about these microbes could therefore be valuable in the case of antibiotic treatment failure.

The pragmatic nature of this study i.e., embedded in routine clinical care hospital practice, the prospective enrolment of patients with community acquired RTIs, application of a stringent case definition of CAP and the analysis of rigorously collected, homogeneous lower respiratory tract samples are major strengths of our work. The study has limitations; the inclusion of a limited number of CAP patients and a short recruitment window during the winter months, suggesting that the findings should be confirmed in further studies. Moreover, patients that were unable to provide a sample from the lower respiratory tract were excluded; resulting in the most severe cases of CAP not being represented, which in turn could explain the low in-hospital mortality rate observed in this study (1%) compared to similar settings^36^.

In conclusion, the use of a rapid syndromic PCR panel for respiratory pathogens was associated with a dramatically reduced time to actionable results and increased detection of clinically relevant pathogens, compared to SDs. A stepwise algorithm for sampling of respiratory specimens with induced sputum and tracheal aspiration was feasible in routine clinical practice and led to obtaining a lower respiratory tract sample in the majority of patients. This suggests that syndromic PCR-based testing may be feasible in acute settings and holds potential to provide clinically actionable results in near real-time. The clinical impact of rapid syndromic pneumonia panels on antibiotic use and patient outcome should therefore be urgently assessed in randomised controlled trials.

## Supplementary Information

Supplementary Information.

## Data Availability

The datasets generated during and/or analysed during the current study are available from the corresponding author on reasonable request.
