# Supplementary material for: Rapid syndromic PCR testing in patients with respiratory tract infections reduces time to results and improves microbial yield
Source: Sci Rep. 2022 Jan 10;12:326. doi: 10.1038/s41598-021-03741-7 (PMC8748978; doi:10.1038/s41598-021-03741-7)
Supplement: Supplementary file 1 — Supplementary Information. [file 41598_2021_3741_MOESM1_ESM.pdf]

Supplementary material

Rapid syndromic PCR testing in patients with respiratory tract infections reduces time to results and improves microbial yield

S. Serigstad[1,2,3], D. Markussen[1]#, H.M.S. Grewal* [3,4], M. Ebbesen[4]#, Ø. Kommedal[3,4], L. Heggelund[3,5], C.H. van Werkhoven[6], D. Faurholt-Jepsen[3,7], T.W. Clark[8], C. Ritz[3,9], E. Ulvestad[3,4], R. Bjørneklett[1,2], S.T. Knoop[1,4] and the CAPNOR study group[§]

Authors affiliations:
[1] Emergency Care Clinic, Haukeland University Hospital, Bergen, Norway
[2] Department of Clinical Medicine, University of Bergen, Bergen, Norway
[3] Department of Clinical Science, Bergen Integrated Diagnostic Stewardship cluster, University of Bergen, Bergen, Norway
[4] Department of Microbiology, Haukeland University Hospital, Bergen, Norway
[5] Department of Internal Medicine, Vestre Viken Hospital Trust, Drammen, Norway.
[6] Julius Center for Health Sciences and Primary Care, University Medical Center Utrecht, Utrecht, The Netherlands.
[7] Department of Infectious Diseases, Rigshospitalet, Denmark.
[8] School of Clinical and Experimental Sciences, Faculty of Medicine, University of Southampton, Southampton, UK
[9] National Institute of Public Health, University of Southern Denmark, Copenhagen, Denmark
# Equal contribution
[§] A complete list of the CAPNOR study group is listed under Consortium.

[*]Corresponding author:
Harleen M.S. Grewal MD, PhD, Professor
Department of Clinical Science, Bergen Integrated Diagnostic Stewardship cluster, University of Bergen, Bergen, Norway
Department of Microbiology, Haukeland University Hospital, Bergen, Norway
Email address: harleen.grewal@uib.no
Phone number: +47 99450554

Postal address:
University of Bergen
Department of Clinical Science,
Faculty of Medicine and Dentistry
The New Lab. Building
5021 Bergen, Norway

Table of contents (page number)

S1: BioFire® FilmArray® Pneumonia panel *plus* (FAP *plus*) targets……………………………………………… 3

S2: Inclusion-/exclusion criteria, and definitions and diagnostic clinical criteria for community acquired pneumonia (CAP)………………………………………………………………………………….. 4

S3: Procedures for sputum induction and endotracheal aspiration…………………………………………… 5

S4: Determining the clinical relevance of microbiological detections………………………………………….. 8

References……… 10

Supplementary material section one (S1)

Supplementary table 1. BioFire® FilmArray® Pneumonia panel *plus* (FAP *plus*) targets

**Bacteria (semi-quantitative)**	**Atypical bacteria (qualitative)**
Acinetobacter calcoaceticus-baumannii complex	*Legionella pneumophila*
Enterobacter cloacae	*Mycoplasma pneumoniae*
Escherichia coli	*Chlamydia pneumoniae*
Haemophilus influenzae	
Klebsiella aerogenes	**Viruses (qualitative)**
Klebsiella oxytoca	Influenza A virus
Klebsiella pneumoniae group	Influenza B virus
Moraxella catarrhalis	Adenovirus
Proteus spp.	Coronavirus
Pseudomonas aeruginosa	Parainfluenza virus 1-4
Serratia marcescens	Respiratory syncytial virus
Staphylococcus aureus	Human rhinovirus/enterovirus
Streptococcus agalactiae	Human metapneumovirus
Streptococcus pneumoniae	Middle East respiratory syndrome coronavirus
Streptococcus pyogenes	
**Antibiotic resistance genes**	
**ESBL:**	
- CTX-M	
**Carbapenemases:**	
- KPC	
- NDM	
- OXA-48-like	
- VIM	
- IMP	
**Methicilin Resistance:**	
- mecA/mecC and MREJ	

Supplementary material section two (S2)

Inclusion- and exclusion criteria

Inclusion criteria:

- Adult aged ≥ 18 years
- Admitted to the emergency department (ED) with suspicion of community acquired pneumonia (CAP)
- Fulfilling at least two of the following criteria: new or worsening cough; new or worsening expectoration of sputum; new or worsening dyspnea; hemoptysis; pleuritic chest pain; radiological evidence of CAP; abnormalities on chest auscultation and/or percussion; fever (≥38.0 ^{o}C).

Exclusion criteria:

- Hospitalization (>48 hours) within the last 14 days prior to admission
- Cystic fibrosis
- Severe bronchiectasis
- A palliative approach (defined as life expectancy below two weeks)
- Patient not willing or able to provide a lower respiratory tract sample

Definitions and diagnostic clinical criteria for CAP. Adapted from Postma D.F. et al. [1]

Clinical CAP:

- Patients with the presence of at least two diagnostic criteria* and in-hospital treatment and/or diagnosis of clinically suspected CAP documented by the treating physician and in agreement with the assessment by a study investigator. In case of disagreement, an additional study investigator will arbitrate.
- Patients with two or more diagnostic criteria and an obvious non-respiratory source of infection are not considered to have a clinical diagnosis of CAP.

Radiologically confirmed CAP:

- Clinical CAP
- The presence of a new or increased infiltrate on chest radiography or computer tomography (CT).

*** Diagnostic criteria:**

- Cough: recent or worsening
- Production of purulent sputum or a change in the character of sputum
- Temperature >38.0°C or <36.1°C
- Auscultatory findings consistent with pneumonia, including rales, evidence of pulmonary consolidation (dullness on percussion, bronchial breath sounds, or egophony), or both
- Leukocytosis (>11.0 × 10^9 white cells per liter or > 8.2 x 10^9 neutrophils)
- C-reactive protein (CRP) level > 50 mg/L
- Dyspnea, tachypnea, or hypoxemia: recent or worsening

Supplementary material section three (S3) [2-5]

Sputum induction by isotonic and/or hypertonic saline

Contraindications

Known airway obstruction due to high humidity or previous serious side effects due to inhalation with saline

Isotonic NaCl (0.9%)

- Patients with spontaneous production and expectoration of sputum
- Always the primary choice in patients with asthma, severe chronic obstructive pulmonary disease (COPD), obstructive airways (prolonged expirium, wheezing sounds), hypoxemia (SpO_2< 92 %) or other known condition with hyper reactive airways
- Contact a physician for evaluation if:
  - Severe COPD (GOLD 3-4) with history of CO_2-retension
- If salbutamol (Ventoline)/ipratropiumbromid (Atrovent) is considered, a physician should be contacted if the patient is:
  - Pregnant
  - Has tachycardia/atrial fibrillation (persistent pulse > 120)

Hypertonic NaCl (1mmol/ml - 5.8 %)

- Patients with little sputum production and little cough
- Contact a physician for evaluation if:
  - Pregnant (**shall not be given to pregnant women**)
  - Obstructive airways (prolonged expirium, wheezing sounds)
  - Known asthma, severe COPD (GOLD 3-4) or other known condition with hyper reactive airways
  - SpO_2 < 92 %
  - Tachycardia/atrial fibrillation (persistent pulse > 120)

Implementation

Use hand disinfection and gloves when handling medications and equipment

- Use droplet precautions
- Only use sterile saline in the nebulizer and check the expiration date
- 5 ml NaCl (either isotonic or hypertonic) is put in the nebulization chamber

- If known asthma, severe COPD (GOLD 3-4), obstructive airways (prolonged expirium, wheezing sounds) or other known condition with hyper reactive airways:
  Use 2.5 ml NaCl and 2.5 ml salbutamol (Ventoline) (2 mg/ml)

- Mouthpiece is the first choice, but masks can also be used. Use nose clip if mouthpiece is used
- Adjust the airflow to 8 liters/minute. Check that the nebulizer is working and producing fine mist
- Observe the patients with regard to worsened respiratory symptoms. If this happens, abort the procedure, contact a physician and start salbutamol (5 mg) and ipratropiumbromid (0.5 mg) in the nebulizer
- If the patients do not provide sputum after the first round of nebulizing, and no side effects are reported, the preceding steps can be repeated by using hypertonic NaCl 1 mmol/ml (5.8%). If hypertonic NaCl was given without any bronchodilator in the first round, please ad salbutamol (Ventoline) in the second round
- Avoid spills or soiling the outside of the container
- Inspect the volume, color and consistence of the material to decide if the test is of good quality
- Clean/disinfect contact points after the procedure

Procedure diagnostic endotracheal aspiration

Equipment

- Face mask with visor/glasses
- Clean gloves
- Wall mounted suction with hose
- Suction catheter with bent tip 14 Ch (green)
- Tracheal mucus extractor
- Lidocain gel
- Sterile container
- 10 mL sterile isotonic saline

Procedure

- The patient should be positioned upright with the head slightly tilted backwards and the chin forwards
- Test the suction
- Connect the tracheal mucus extractor
- Use lidocaine gel at the tip of the catheter and in the patient's nose
- Do not touch the distal 15 cm of the catheter
- The catheter is inserted through the nose and down to the hypopharynx without suction, until respiratory sounds are audible through the catheter end
- The patient is then instructed to cough or take a deep breath, while the catheter is lead 3-5 cm further down into the trachea
- The catheter is then connected to the tracheal mucus extractor
- Start suction, but no more than 15 seconds at a time and without moving the catheter
- The catheter is retracted slowly with a rotating movement, without suction
- After the procedure, if necessary, the catheter can be flushed with 1-2 mL sterile isotonic saline to collect any remaining respiratory specimens into the sample container

Supplementary material section four (S4)

Determining the clinical relevance of microbiological detections [6-23]

1. **Clinically relevant**

   A. **Proven etiology**

   Diagnosis of CAP AND

   i. Detection of the same bacteria in the respiratory tract specimen and blood culture
   ii. *Streptococcus pneumoniae* detected in respiratory tract specimen and positive pneumococcal urine antigen test
   iii. Detection of *C. pneumoniae*, *B. pertussis*, *L. pneumophilia* or *M. pneumoniae* in respiratory tract specimen
   iv. Positive urine antigen test for *L. pneumophilia*
   v. Growth of category B- or C-bacteria (Supplementary table 2) in blood culture and no other detectable focus of infection

   B. **Probable etiology**

   Diagnosis of CAP AND

   i. Detection of category B-bacteria in a respiratory tract specimen
   ii. Detection of category C-bacteria in respiratory tract specimens in a patient with chronic underlying disease[a] and/or patients with a recent history of antibiotic use[b]
   iii. Detection of a single species of category D-bacteria in a respiratory tract specimen in immunocompromised patients[c] and/or patients with a recent history of antibiotic use or other known risk of infections due to certain bacteria[d]
   iv. Detection of category D-bacteria in blood culture and no other detectable focus of infection
   v. Positive pneumococcal urine antigen test

2. **Uncertain relevance**

   i. All detections not defined by the above criteria.

---

[a] Such as chronic lung disease, diabetes mellitus, chronic kidney disease, immunosuppression
[b] Two courses of antibiotics in the last 6 months
[c] Chronic lung disease, except for asthma, acquired or congenital immunodeficiency, including HIV-infection, chronic use of corticosteroids > 7,5 mg prednisolone or equipotent dosage, use of immunosuppressants (ATC L04), ongoing treatment with cytostatic (ATC L01).
[d] >Prior infection with resistant microbes (e.g., extended spectrum beta-lactamase (ESBL), Methicillin-resistant Staphylococcus aureus (MRSA).

Supplementary table 2. Respiratory tract microbes divided in different categories according to their potential clinical relevance

**Category**	**Microbe**
**A: Always pathogens**<br>Always considered relevant in a patient with respiratory tract infection	Adenovirus [9,11,15,17]<br>Coronavirus [11,15,17]<br>Human metapneumovirus [9,15,17]<br>Influenza virus [9,11,15,17]<br>MERS [12]<br>Parainfluenza virus [11,15,17]<br>Rhinovirus [9,15,17]<br>RS-virus [9,11,15,17]<br><br>*Bordetella pertussis* [9]<br>*Chlamydia pneumoniae* [6,9,11,15,17]<br>*Legionella pneumophila* [6,9,17]<br>*Mycoplasma pneumoniae* [6,9,11,15,17]
**B: Usually pathogens**<br>Can be colonizers, but are usually considered relevant in a patient with pneumonia	*Haemophilus influenzae* [6,9,11,15,17]<br>*Streptococcus pneumoniae* [6,9,11,15,17,23]<br>*Streptococcus pyogenes* [14,21,23]
**C: Usually not pathogens**<br>Usually colonizers, but can cause pneumonia, especially in patient with chronic diseases	*Klebsiella pneumoniae* [10,17,23]<br>*Moraxella catarrhalis* [6,8,15,17]<br>*Staphylococcus aureus* [6,15,17,18,23]
**D: Usually not pathogens**<br>A seldom cause of pneumonia in otherwise healthy patients. May be considered relevant if detected as the only bacterial species in a patient with immunosuppression and/or several previous courses of antibiotics	*Acinetobacter calcoaceticus-baumannii-complex* [7,15,23]<br>*Enterobacter cloacae* [9,15,22]<br>*Escherichia coli* [9,15,17,22,23]<br>*Klebsiella aerogenes* [9,15]<br>*Klebsiella oxytoca* [9,15]<br>*Proteus species* [9,15,22]<br>*Pseudomonas aeruginosa* [15-17,22,23]<br>*Serratia marcescens* [9,15,22]<br>*Streptococcus agalactiae* [19]

References

1 Postma, D. F. *et al.* Antibiotic treatment strategies for community-acquired pneumonia in adults. *N Engl J Med* **372**, 1312-1323, doi:10.1056/NEJMoa1406330 (2015).

2 Paggiaro, P. L. *et al.* Sputum induction. *Eur Respir J Suppl* **37**, 3s-8s, doi:10.1183/09031936.02.00000302 (2002).

3 Cataldo, D. *et al.* Induced sputum: comparison between isotonic and hypertonic saline solution inhalation in patients with asthma. *Chest* **120**, 1815-1821, doi:10.1378/chest.120.6.1815 (2001).

4 *Tuberculosis guidelines from the Norwegian Institute of Public Health*, <http://www.fhi.no/artikler/?id=83629> (last accessed 21st October 2021)

5 *National evidence based guidelines database by the Norwegian Institute of Public Health*, <https://www.helsebiblioteket.no/fagprosedyrer/ferdige/indusert-sputum> (last accessed 21st October 2021)

6 Apisarnthanarak, A. & Mundy, L. M. Etiology of community-acquired pneumonia. *Clin Chest Med* **26**, 47-55, doi:10.1016/j.ccm.2004.10.016 (2005).

7 Asai, N. *et al.* Clinical manifestations and risk factors of community-onset Acinetobacter species pneumonia in Japan; case control study in a single institute in Japan. *J Infect Chemother* **25**, 639-642, doi:10.1016/j.jiac.2019.03.016 (2019).

8 Hirai, J. *et al.* Clinical characteristics of community-acquired pneumonia due to Moraxella catarrhalis in adults: a retrospective single-centre study. *BMC Infect Dis* **20**, 821, doi:10.1186/s12879-020-05564-9 (2020).

9 Holter, J. C. *et al.* Etiology of community-acquired pneumonia and diagnostic yields of microbiological methods: a 3-year prospective study in Norway. *BMC Infect Dis* **15**, 64, doi:10.1186/s12879-015-0803-5 (2015).

10 Lin, Y. T., Jeng, Y. Y., Chen, T. L. & Fung, C. P. Bacteremic community-acquired pneumonia due to Klebsiella pneumoniae: clinical and microbiological characteristics in Taiwan, 2001-2008. *BMC Infect Dis* **10**, 307, doi:10.1186/1471-2334-10-307 (2010).

11 Mandell, L. A. Community-acquired pneumonia: An overview. *Postgrad Med* **127**, 607-615, doi:10.1080/00325481.2015.1074030 (2015).

12 Memish, Z. A., Perlman, S., Van Kerkhove, M. D. & Zumla, A. Middle East respiratory syndrome. *Lancet* **395**, 1063-1077, doi:10.1016/S0140-6736(19)33221-0 (2020).

13 Metlay, J. P. *et al.* Diagnosis and Treatment of Adults with Community-acquired Pneumonia. An Official Clinical Practice Guideline of the American Thoracic Society and Infectious Diseases Society of America. *American Journal of Respiratory and Critical Care Medicine* **200**, e45-e67, doi:10.1164/rccm.201908-1581ST (2019).

14 Muller, M. P. *et al.* Clinical and epidemiologic features of group a streptococcal pneumonia in Ontario, Canada. *Arch Intern Med* **163**, 467-472, doi:10.1001/archinte.163.4.467 (2003).

15 Musher, D. M. & Thorner, A. R. Community-Acquired Pneumonia. *New England Journal of Medicine* **371**, 1619-1628, doi:10.1056/NEJMra1312885 (2014).

16 Restrepo, M. I. *et al.* Burden and risk factors for Pseudomonas aeruginosa community-acquired pneumonia: a multinational point prevalence study of hospitalised patients. *Eur Respir J* **52**, doi:10.1183/13993003.01190-2017 (2018).

17 Rider, A. C. & Frazee, B. W. Community-Acquired Pneumonia. *Emerg Med Clin North Am* **36**, 665-683, doi:10.1016/j.emc.2018.07.001 (2018).

18 Self, W. H. *et al.* Staphylococcus aureus Community-acquired Pneumonia: Prevalence, Clinical Characteristics, and Outcomes. *Clin Infect Dis* **63**, 300-309, doi:10.1093/cid/ciw300 (2016).

19 Skolnik, K. *et al.* Group B streptococcus (GBS) is an important pathogen in human disease-but what about in cystic fibrosis? *BMC Infect Dis* **17**, 660, doi:10.1186/s12879-017-2729-6 (2017).

20 Stralin, K., Olcen, P., Tornqvist, E. & Holmberg, H. Definite, probable, and possible bacterial aetiologies of community-acquired pneumonia at different CRB-65 scores. *Scand J Infect Dis* **42**, 426-434, doi:10.3109/00365540903552353 (2010).

21 Tamayo, E., Montes, M., Vicente, D. & Pérez-Trallero, E. Streptococcus pyogenes Pneumonia in Adults: Clinical Presentation and Molecular Characterization of Isolates 2006-2015. *PLoS One* **11**, e0152640, doi:10.1371/journal.pone.0152640 (2016).

22 von Baum, H., Welte, T., Marre, R., Suttorp, N. & Ewig, S. Community-acquired pneumonia through Enterobacteriaceae and Pseudomonas aeruginosa: Diagnosis, incidence and predictors. *Eur Respir J* **35**, 598-605, doi:10.1183/09031936.00091809 (2010).

23 Wongsurakiat, P. & Chitwarakorn, N. Severe community-acquired pneumonia in general medical wards: outcomes and impact of initial antibiotic selection. *BMC Pulm Med* **19**, 179, doi:10.1186/s12890-019-0944-1 (2019).
